# Inadequate Reporting of Cointerventions, Other Methodological Factors, and Treatment Estimates in Cardiovascular Trials: A Meta-Epidemiological Study

**DOI:** 10.1016/j.mayocpiqo.2023.04.010

**Published:** 2023-06-02

**Authors:** Jonas Bührer, Cinzia Del Giovane, Baris Gencer, Luise Adam, Christina Lyko, Martin Feller, Bruno R. Da Costa, Drahomir Aujesky, Douglas C. Bauer, Nicolas Rodondi, Elisavet Moutzouri

**Affiliations:** a Institute of Primary Health Care (BIHAM), University of Bern, Switzerland; b Department of General Internal Medicine, Inselspital, University Hospital of Bern, University of Bern, Switzerland; c Department of Cardiology, University of Geneva, Switzerland; d Applied Health Research Centre (AHRC), Li Ka Shing Knowledge Institute of St. Michael’s Hospital, Institute of Health Policy, Management, and Evaluation, University of Toronto, Ontario, Canada; e Departments of Medicine and Epidemiology and Biostatistics, University of California, San Francisco

## Abstract

**Objective:**

To assess how inadequate reporting of cointerventions influences estimated treatment effects in recent cardiovascular trials.

**Methods:**

Medline/Embase were systematically searched from January 1, 2011 to July 1, 2021 for trials evaluating pharmacologic interventions on clinical cardiovascular outcomes published in 5 high-impact journals. Information on adequate vs inadequate reporting of cointerventions, blinding, risk of bias due to deviations of intended interventions (low vs high/some concerns), funding (nonindustry vs industry), design (superiority vs noninferiority), and results were assessed by 2 reviewers. The association with effect sizes was assessed using meta-regression random-effect analysis, expressed as ratios of odds ratios (ROR). RORs of >1.0 indicated that trials with the methodological factor pointing to lower quality report larger treatment estimates.

**Results:**

In total, 164 trials were included. Of the 164 trials, 124 (74%) did not adequately report cointerventions; 89 of the 164 trials (54%) provided no information regarding cointerventions, and 70 of the 164 (43%) were at risk of bias due to inadequate blinding. Moreover, 86 of the 164 (53%) were at risk of bias due to deviation of intended interventions. Of the 164 trials, 144 (88%) were funded by the industries. Trials with inadequate reporting of cointerventions had larger treatment estimates for the primary end point (ROR, 1.08; 95% CI, 1.01-1.15; *I*^*2*^=0%). No significant association with results for blinding (ROR, 0.97; 95% CI, 0.91-1.03; *I*^*2*^=66%), deviation of intended interventions (ROR, 0.98; 95% CI, 0.92-1.04; *I*^*2*^=0%), or funding (ROR, 1.01; 95% CI, 0.93-1.09; *I*^*2*^=0%) was found.

**Conclusion:**

We conclude that trials with inadequate reporting of cointerventions showed larger treatment effect estimates, potentially indicating overestimation of therapeutic benefit.

**Trial Registration:**

Prospero Identifier: CRD42017072522

Randomized controlled trials (RCTs) are expected to provide the highest level of evidence regarding the effects of a therapeutic intervention,^1^^,^^2^ but their results are subject to potential biases. Bias can occur in one or several stages of an RCT, for example, on randomization, data collection, during follow-up, or outcome assessment, and may take several forms. Previous studies have shown an association of inadequate allocation generation or concealment with larger treatment effect estimates, particularly in trials with subjective outcomes.^1^^,^^3^ Trial results can also be biased by inadequate blinding of participants, health care providers, or outcome assessors.^4^^,^^5^ Funding and industry sponsorship may also introduce bias. The effects of these factors have been assessed in previous studies, but results have been inconsistent.^1^^,^^3^^,^^4^^,^^6^, ^7^, ^8^, ^9^, ^10^, ^11^

Performance bias may arise during follow-up if participants receive unbalanced care (such as cointerventions) after randomization.^9^^,^^12^, ^13^, ^14^, ^15^, ^16^, ^17^, ^18^, ^19^ Outcomes in cardiovascular RCTs depend on the individual cardiovascular risk of participants and the treatment initiated during the trial, for instance, to treat high blood pressure, diabetes, or dyslipidemia. Thus, in a cardiovascular RCT, a cointervention is an additional treatment that a patient may receive before the incidence of the primary end point that modifies participant’s cardiovascular risk and affect the outcome of the trial. For example, in the Women’s Health Initiative (WHI),^13^ which examined the effect of hormone therapy on cardiovascular outcomes, it was shown that differential use of statins has significantly influenced the effects on coronary artery disease and stroke and, thus, may have confounded the results (23.6% of participants assigned to placebo and 18.2% assigned to the intervention group at 6 years reported statin use).^13^ In HERS (Heart and Estrogen/progestin Replacement Study), the hazard ratio for coronary artery disease in the active group vs placebo was 0.99 (95% CI, 0.79-1.24) and 0.96 (95% CI, 0.77-1.29) after the adjustment for postrandomization statin use (22% vs 18% in the placebo vs active group reported statin use).^13^ In another double-blinded RCT designed to test the effects of fenofibrate vs placebo on hard cardiovascular end points, 17% of the participants on placebo were treated with statins vs 8% in the fenofibrate group, leading to unbalanced cointerventions and a possible bias of the results toward the null, which might have masked a moderately larger treatment benefit.^17^ We have recently found that approximately two-thirds of recent cardiovascular trials failed to adequately report cointerventions, independent of blinding status.^20^ However, the influence of blinding and cointerventions on effect sizes of recent cardiovascular RCTs has not been previously examined.

Thus, we set out to systematically examine the methodological quality factors associated with increased effect sizes in recent cardiovascular RCTs. We estimated the effect of cointerventions, blinding, bias due to deviation of intended intervention, funding, and study design on the results of RCTs. A secondary objective was to detect spin, defined as misleading reporting, interpretation, or extrapolation of study results.

## Methods

### Eligibility, Information Source, and Article Selection

This work continues our earlier study on reporting of cointerventions in cardiovascular trials,^20^ so we summarize our methods and refer to this publication where appropriate.

We searched Medline and Embase for RCTs that evaluated pharmacologic interventions on binary cardiovascular outcomes as primary outcomes (fatal and/or nonfatal myocardial infarction, fatal and/or nonfatal stroke, mortality, and their composite outcomes), published in the 5 highest impact general medical journals (*New England Journal of Medicine*, *Lancet*, *Journal of the American Medical Association*, *British Medical Journal*, and *Annals of Internal Medicine*) between 2011 and 2021. (See Supplemental Table 2, available online at http://www.mcpiqojournal.org, for details of our search strategy with last search on July 27, 2021). We also hand-searched the online library of these 5 journals. One reviewer (E.M.) screened all titles and abstracts and identified relevant trials. A second reviewer (J.B.) assessed eligible abstracts. We conformed to the PRISMA guidelines for reporting systematic reviews and meta-analyses^21^ and guidelines for reporting meta-epidemiological methodology research.^22^ We registered our protocol on PROSPERO (CRD42017072522).

### Data Extraction, Definitions, and Types of Methodological Features

We retrieved trial publications in English from January 2011 through July 2021 and analyzed their full text, extracting not only all available information from the original trial reports but also, where available, supplementary material and protocols. Four reviewers (C.L., L.A., E.M., J.B.), who are trained physicians and researchers, independently extracted data into a prespecified extraction form. They resolved disagreement by discussion or called in a third researcher. Reviewers retrieved the following information: basic trial characteristics (journal, publication year, and clinical area of interest); study design (superiority vs noninferiority); type of intervention and comparator; outcomes; number of participants and number of events in each group; follow-up duration; information regarding methods of blinding participants, health care providers and outcome assessors; information about cointerventions; implementation of study treatment; adherence to study treatment; crossovers; type of statistical analysis; and funding source (industry/nonindustry). If data were missing, we did not contact the authors.

Strict criteria were used to decide whether cointerventions were “adequately” or “inadequately” reported.^20^ We looked for the following concomitant medications: statins, antihypertensive drugs, or antiplatelets over the postrandomization period until patients have been censored or have reached the primary outcome. Furthermore, in trials with diabetic participants, in the definition of cointerventions, antidiabetic drugs were also included. Similarly, anticoagulants were included in trials comprising patients with an indication to be treated with anticoagulants (eg, atrial fibrillation or mechanical valves). We also defined 2 special categories of cointerventions as follows: (1) in RCTs where there was an index procedure after randomization, in addition to concomitant medications (statins, antihypertensive drugs, and antiplatelets) over the follow-up, procedural characteristics and periprocedural medications between the groups would also be cointerventions; and (2) in RCTs with an index procedure after randomization but with a follow-up of less than 1 month, cointerventions would be procedural characteristics and periprocedural medications without considering concomitant medications (statins, antihypertensive drugs, and antiplatelets). If the trials reported all 3-5 medications of interest (reported as percentages or absolute numbers for both groups separately), we defined it as “adequately” reported. Alternatively, the authors should have stated explicitly that cointerventions defined as the medications of interest were balanced between the groups. Although advice for smoking, diet, and physical activity are also effective cointerventions, they are difficult to quantify, are rarely assessed in the original studies, and, therefore, not evaluated in this study.^20^

Blinding was defined as an absence of awareness by participants or health care providers of the intervention status of individual participants throughout the trial. We classified trials as adequately/inadequately blinded, according to Cochrane Collaboration risk of bias tool 2011, as previously described.^20^ We used the Cochrane risk of bias in randomized trials tool (RoB2 tool) to assess risk of bias caused by deviations from intended interventions, which also implements adherence to trial medication. Trials were classified as “high risk of bias”, “some concerns”, or “low risk of bias”.^19^ Trials classed as “some concerns” and “high risk of bias” were grouped for the analysis. Funding was divided into “industry” and “nonindustry” (we particularly checked whether an industry was involved in any step of the trial design, conduction, or analysis: if, for example, a drug was provided free of charge but the industry was not involved in any step of the design, conduction, or analysis, it was defined as “nonindustry”; if multiple funding sources were noted and one of them was an industry involved in any step of the design, conduction, or analysis, it was defined as industry funded).^20^ Spin was defined as the “use of specific reporting strategies, from whatever motive, to highlight that the experimental treatment is beneficial, despite a statistically nonsignificant difference for the primary outcome (ie, inappropriate use of causal language), or to distract the reader from statistically nonsignificant results (ie, to focus on a statistically significant secondary result).”^23^

### Data Analysis

We used a meta-epidemiological approach,^24^ to assess the association between reporting cointerventions (adequate vs inadequate), blinding (adequately vs inadequately blinded), risk of bias according to deviations of intended interventions (“at low risk of bias” vs trials “at risk of bias”), funding (nonindustry funded vs industry funded), and positive results. First, we identified trials with the same medication group (to included homogeneous sets of trials) and conducted random-effects meta-analyses for the composite end points within each set of trials. Because studies reported different effect sizes (eg, relative risks, hazard ratios, and odds ratios [ORs]), first, we modeled all effect estimates as ORs; then, outcomes were coded so that an OR less than 1 indicates a beneficial effect of the experimental intervention, as previously described (see Supplemental Table 1, available online at http://www.mcpiqojournal.org, for corresponding effect sizes as reported in the original publication and OR as calculated through random-effects meta-analysis, categorized according to adequate reporting of cointervention vs not).^1^^,^^3^^,^^4^^,^^24^^,^^25^ Then, to explore effect-measure modification, we performed a meta-regression analyses in which the independent variable was the quality characteristic, the coefficient represented the ratio of odds ratio (ROR) = OR_studies at low risk of bias_/OR_studies at risk of bias_. An ROR of >1 indicates larger effect estimates in trials characterized, with the methodological factor pointing to a higher risk of bias. Heterogeneity was measured by using the *I*^*2*^ statistic (0% to 40%: not important heterogeneity; 30% to 60%: moderate heterogeneity; 50% to 90%: substantial heterogeneity; 75% to 100%: considerable heterogeneity).^26^ Three-arm trials were included in the literature search but only the results of one experimental intervention vs placebo were included in the analysis. In case of 2 or multiple primary outcomes, we chose according to the following: (1) the primary outcome that was also reported in the protocol, if a protocol was available and (2) the composite primary outcome that was most close to our defined outcome: fatal and/or nonfatal myocardial infarction, fatal and/or nonfatal stroke, mortality, and their composite outcomes.

We further performed multivariable adjustment for other methodological factors and study-level variables (blinding, study design, and funding) and performed sensitivity analyses by excluding very potent drugs and outliers (eg, trials with early stop). Because almost all of our studies (excluding n=3) were large studies with more than 800 participants, we did not adjust for study sample size.

*P* values were 2-sided and considered significant at *P*<.05. We used Stata version 16.0 for data management, analysis, and graphics.

## Results

### Trial Characteristics and Descriptive Results of Trials

Our literature search identified 1901 potentially eligible reports. After screening titles and abstracts, we evaluated 200 full-text articles; 164 were included in the analysis (Supplemental Figure 1, available online at http://www.mcpiqojournal.org). The main reason trials were excluded was not being an RCT design. Of the trials we included, 108 (66%) trials were published in the *New England Journal of Medicine*, 31 (19%) in *Journal of the American Medical Association*, and 22 (13%) in *Lancet*; 124 (76%) trials had a superiority design; 144 (88%) were industry sponsored; 91 (55%) trials used a placebo as a comparator; 63 (38%) trials studied antiplatelet/anticoagulant drugs, 25 (15%) studied antidiabetic drugs, 19 (12%) were lipid-modifying trials. Nine of the 164 trials recorded 2 coprimary outcomes.

Of the 164 trials, 124 (74%) did not adequately report cointerventions; of which 89 (54% of all trials) provided no information regarding cointerventions; 70 of the 164 (43% of all trials) were at risk of bias due to inadequate blinding, of which 34 (21% of all trials) were at high risk. Moreover, 49 of the 164 (30% of all trials) were at high risk of bias due to deviation of intended interventions; 37 (23% of all trials) showed some concerns; and 78 (47% of all trials) were at low risk of bias. Of the 164 trials, 144 (88% of all trials) were industry funded. Furthermore, 95 (58%) provided information regarding medication adherence and had sufficient adherence (>80% of patients being adherent to trial medication intake); 12 (7%) provided no information on medication adherence; and 57 (35%) trials reported insufficient adherence to the trial medication (<80%). Table 1 summarizes trial characteristics according to the reporting of cointerventions. Characteristics were mostly balanced between the trials reporting vs not adequately reporting cointerventions. However, industry-sponsored RCTs most often did not report cointerventions. For medication categories, antidiabetic drug trials reported more cointerventions, whereas category “various” most often did not report cointerventions adequately. We identified 23 (14%) trials with a spin where reporting and interpretation of outcomes was inconsistent with trial results.

**Table 1:** Trial Characteristics (N=164) According to Cointerventions Reporting

Variables	Cointerventions reported (n=42), n (%)	Cointerventions not adequately reported (n=122), n (%)
Journal		
New England Journal of Medicine	30 (71.4)	78 (63.9)
Lancet	7 (16.7)	15 (12.3)
Journal of the American Medical Association	5 (11.9)	26 (21.3)
British Medical Journal	—	1 (0.8)
Annals of Internal Medicine	—	2 (1.6)
Type of comparator		
Placebo	22 (76.2)	93 (76.2)
Active	10 (23.8)	29 (23.8)
Trial design		
Superiority	11 (26.2)	29 (23.8)
Noninferiority/equivalence	31 (73.8)	93 (76.2)
Type of funding source		
Industry sponsored	28 (66.7)	104 (85.2)
Nonindustry	14 (33.3)	18 (14.8)
Type of intervention^a^		
Antihypertensives/diuretics/heart failure treatments	3 (7.14)	12 (9.8)
Antiplatelets/anticoagulants	19 (45.2)	44 (36.1)
Lipid-modifying medications	5 (11.9)	11 (11.5)
Antidiabetics	10 (23.8)	15 (12.3)
Antiinflammatory, antirheumatic, antineoplastic	1 (2.4)	9 (7.4)
Cardiac therapy^b^	0 (0)	6 (4.9)
Various^c^	4 (9.5)	22 (18.0)

a Classified according to the ATC codes; for detailed description of the included trials, see Supplemental Table 2.

b Cardiac therapy included antianginal treatment and antiarrhythmic medications.

c Various includes antiobesity preparations, medications for treating bone disease, vitamins, and combination of different treatments.

### Effect on Treatment Estimates

**Figure 1:**
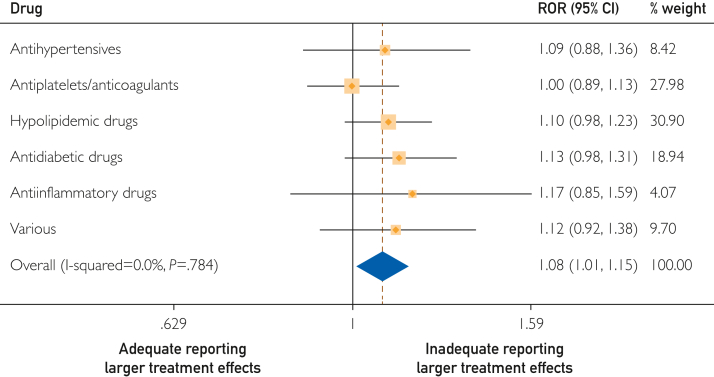
Forest plot on the association of cointerventions and treatment effect estimates (n=162).^a^ Ratio of odds ratio (ROR) >1.0, indicating larger effect estimates in studies with inadequate reporting of cointerventions. ^a^2 trials did not provide number of events for primary outcome and were, therefore, excluded. “Various” includes cardiac therapy (antianginal and antiarrhythmics), antiobesity preparations, medications for treating bone disease, vitamins, and combination of different treatments.

**Figure 2:**
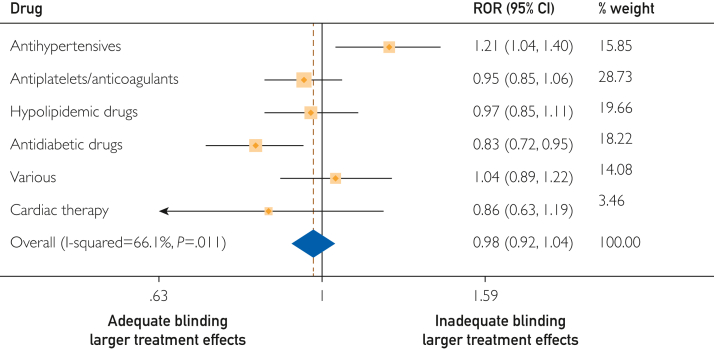
Forest plot of the association of risk of bias due to inadequate blinding and treatment effect estimates (n=162).^a^ Ratio of odds ratio (ROR) >1.0, indicating larger effect estimates in studies with inadequate blinding of participants and/or personnel. ^a^2 trials did not provide number of events for primary outcome and were, therefore, excluded. “Various” includes antiobesity preparations, medications for treating bone disease, vitamins, and combination of different treatments.

In the meta-analytic analysis, the association between inadequate reporting of cointerventions and effect estimates was 1.08 (95% CI, 1.01-1.15), expressed as an ROR, indicating that trials that inadequately reported cointerventions showed larger effect estimates (Figure 1). For blinding, the ROR was 0.97 (95% CI, 0.91-1.03) when we compared trials that were adequately and inadequately blinded (Figure 2). The ROR was 0.97 (95% CI, 0.91-1.03) for risk of bias due to deviations from intended interventions and 1.01 (95% CI, 0.93-1.09) for industry funding (Supplemental Figures 2 and 3, available online at http://www.mcpiqojournal.org). Supplemental Table 1 (available online at http://www.mcpiqojournal.org) lists all included RCTs with effect sizes calculated as OR vs effect sizes as published according to the reporting of cointerventions.

### Sensitivity and Subgroup Analyses

Our main results remained the same when we adjusted all our analyses for the type of study (superiority vs noninferiority), blinding, or industry funding (Table 2).

**Table 2:** Meta-Regression Results on the Association of Cointervention Reporting with Treatment Estimates: Sensitivity Analyses^a^^,^^b^

Sensitivity analysis	N^c^	ROR (95% CI)
All RCTs per groups adjusting for the type of study (superiority, noninferiority)	162	1.08 (1.02-1.15)
All RCTs per groups adjusting for the risk for bias due to unblinding	162	1.07 (1.00-1.14)
All RCTs per groups adjusting for funding	162	1.10 (1.02-1.18)
All RCTs per groups after dropping highly potent drugs	157	1.07 (1.01-1.13)
All RCTs per groups after dropping antidiabetic trials stopped early	160	1.07 (1.00-1.14)
All RCTs per groups after dropping antihypertensive trials stopped early	159	1.07 (1.01-1.14)

a RCT, randomized controlled trial.

b Ratio of odds ratio (ROR) >1.0, indicating larger effect estimates in trials not adequately reporting cointerventions.

c Two trials did not provide the number of events for primary outcome and were, therefore, excluded.

Results for cointerventions were consistent in the sensitivity analysis that excluded trials with highly potent hypolipidemic drugs (trials with PCSK9-inhibitors, statins, and icosapent ethyl) (Table 2). In individual medication categories, the association of cointerventions were almost similar in all groups, with the largest seen for hypolipidemic (ROR, 1.10; 95% CI, 0.98-1.23) and antidiabetic (ROR, 1.13; 95% CI, 0.98-1.31) drugs (Figure 1).

For the risk of bias due to inadequate blinding, 2 subgroups showed statistically significant and heterogenic results, which explains the *I*^*2*^ of 66.1% (Figure 2). For antihypertensive medications, the ROR was 1.22 (95% CI, 1.05-1.42), indicating that inadequate blinding increases treatment estimates (Figure 2). For antidiabetic drugs, the ROR was 0.83 (95% CI, 0.72-0.95). However, by performing additional sensitivity analyses dropping 2 antidiabetic trials stopped early for benefit according to prespecified rules (PMID 30990260 and 32970396), results for antidiabetic drugs were attenuated and no longer significant for risk of bias due to inadequate blinding, whereas risk of bias associated with cointerventions was not affected (ROR for risk of inadequate blinding in antidiabetic drugs, 0.87; 95% CI, 0.75-1.01). Results in the category of antihypertensive drugs remained significant even after dropping 3 trials stopped early for benefit according to prespecified rules (PMID 25176015, 21073363, and 26551272; ROR for risk of inadequate blinding for antihypertensive drugs, 1.20; 95% CI, 1.08-1.33), whereas the risk of bias associated with cointerventions was not affected (Table 2).

## Discussion

In this systematic review and meta-epidemiological study of recent large cardiovascular RCTs, trials with inadequate reporting of cointerventions showed, on average, larger treatment estimates compared with trials with adequate reporting of cointerventions, with an increase odds of treatment benefit of 8% for the primary end point. No consistent evidence was found for larger treatment estimates in trials with inadequate blinding of participant and/or personnel or at risk of bias caused by deviations from intended interventions or industry funding.

Postrandomization bias in randomized trials may be caused by cointerventions; if they are unbalanced between trial groups and affect the outcome, this could lead to bias and exaggerate or reduce treatment estimates. Few studies have sought to determine the extent to which unbalanced cointerventions could change the results of an RCT. One meta-epidemiological study tried to address the effect of “similarity of cointerventions” on effect sizes in 3 data sets,^15^ but results were inconsistent. Only the third data set, which used dichotomous outcomes (vs continuous outcomes), showed an effect. This previous study found that trials reporting similar cointerventions or no cointerventions recorded larger treatment effect estimates than trials that did not report on similar cointerventions.^15^ However, this study did not report in which category the “not reporting” of cointerventions was classified or how “cointerventions” were assessed; moreover, the studies included were very old (published between 1960 and 1995), whereas only 3 studies were on “circulatory” diseases (2 with only mortality as an outcome and 1 with deep vein thrombosis). Evidence from single studies suggests that cointerventions such as statins or antihypertensive drugs may influence treatment estimates.^12^^,^^13^^,^^17^ In WHI and HERS, a difference of up to 5% in the use of statins between groups influenced the effect estimates.^13^ In our study, three-fourths of trials inadequately reported cointerventions, so we could not explore the effects of balanced and unbalanced cointerventions. For cointerventions to have an effect on outcomes, the following conditions are required minimally: (1) substantial number of patients exposed to cointerventions and (2) cointerventions need to be unidirectional. Although we could not assess these conditions due to not reporting, we did find that trials that inadequately reported cointerventions were associated with exaggerated treatment effect estimates. An explanation for the observed association could be that deviating from protocol and failing to report cointerventions may serve as a marker of lower study quality, indicating larger effect estimates in poor quality studies, as previously reported.^15^ One previous study has shown that inadequate blinding was associated with an increased risk for cointerventions,^27^ so we would have expected that the effect of not reporting cointerventions would be at least associated with inadequate blinding.^15^ A possible explanation could be that risk of nonblinding cannot be properly detected, as previously published.^9^ Our results support arguments that cointervention reporting should be standardized and, where applicable, consider them in the statistical analysis if needed. Possible cointerventions should be identified while drafting the protocol. We laid groundwork by exploring the possible effect of cointerventions on trial results and encourage future researchers to design studies that will help us better understand this association.

Lack of blinding is usually considered a source of bias, although results from meta-epidemiological studies are contradictory.^1^^,^^3^^,^^6^^,^^25^^,^^28^ Previous studies have shown that estimated bias in intervention effects caused by inadequate blinding varied across RCTs by the type of outcome.^1^^,^^3^^,^^6^^,^^25^^,^^28^ A recent meta-epidemiological study also found no evidence that estimated treatment effects differed between RCTs that blinded or did not blind participants, health care providers, or outcome assessor; this was also true for trials with subjective outcomes.^4^ Other recently published meta-epidemiological studies did not confirm these findings—particularly in trials with subjective outcomes.^5^^,^^11^ Overall, we found no strong evidence that treatment estimates differed between trials with adequate and inadequate blinding. However, in the category of antihypertensive drugs, trials at risk of inadequate blinding showed larger effect estimates, and this association should be further investigated. In this systematic review, no study was at a risk of bias because of its randomization process or nonblinding of outcome assessors.

We were guided by the Cochrane risk of bias tool 2 to assess risks of bias due to deviations from intended interventions, which also assesses the adherence to the intervention. We found no evidence that this risk of bias was associated with larger effect estimates.

A third of the included studies were of a noninferiority design. The limitations of noninferiority trials are well known and discussed extensively in the literature.^10^^,^^29^ Industry-sponsored trials are more likely to report favorable results, particularly when conducted in a noninferiority design, independent of medical domain.^10^^,^^30^ After including these variables in the multivariable-adjusted meta-regression, the main results did not change. Most recent RCTs were industry sponsored; 88% of trials were industry funded in our study. With an ROR of 1.01, we did not find an association between industry involvement and effect sizes. A systematic review from 2006 showed that cardiovascular trials funded by the industry reported a markedly higher number of positive results, but no analysis was conducted to determine the association of industry funding with treatment estimates.^7^ It is possible we missed this association because our comparator group (trials without industry funding) was underpowered in our analysis. Only 12% of trials were not industry funded, evident in the wide confidence intervals, for example, in the antidiabetic drug subgroup.

Among trials with statistically nonsignificant primary outcomes, we identified 23 (14%) trials with a spin, defined as inconsistent reporting or interpretation of trial results. A recent published work identified spin in 57% of the abstracts and 67% of the main texts of cardiovascular trials published in 6 high-impact journals.^31^ The difference in spin prevalence may be because of our selection criteria since we only included medication trials with hard cardiovascular outcomes published in general medical journals.

Our study has limitations. We confined our study to cardiovascular trials published in major medical journals in an effort to reduce incomplete reporting, but even so, trial reports are sometimes incomplete.^32^ The heterogeneity of trials may have limited our meta-analytic approach, although we tried to reduce heterogeneity by analyzing trials by medication category and by including larger trials with similar binary objective outcomes. In addition, previous meta-epidemiological studies which included high impact factor publications used similar study methodology as ours.^24^ Drugs tested in trials with inadequate reporting of cointerventions may have been less potent, although we see no reason why reporting of cointerventions should be different between effective and less-effective drugs, and our results for cointerventions were consistent in the sensitivity analysis that excluded the trials of highly potent drugs. We acknowledge that ROR as a measure of effect may be biased; however, this may mostly be the case when, for example, results are inverted for some clinical questions: in our case, we did not apply a selective inversion rule.^33^ Furthermore, our study sample is smaller than those of previous meta-epidemiological studies,^4^ which may have limited our analysis in medication categories, including very few studies with a specific characteristic. Our results pose a risk of ecologic fallacy: inadequate reporting of cointerventions could be a marker of poor study quality.

Future research should explore how inadequate reporting of cointerventions and unbalanced cointerventions and potentially exaggerated effect estimates are associated within meta-analyses, with larger sample sizes and exploring other medical fields too.

## Conclusion

In this meta-epidemiological study of recent large cardiovascular RCTs, no strong association between treatment estimates and blinding of participants and or/personnel was found. However, inadequate reporting of cointerventions was associated with potentially exaggerated effect estimates that may indicate therapeutic benefits are overestimated. Cardiovascular trials should systematically report cointerventions and adjust the analyses for this possible bias.

## Potential Competing Interests

The authors report no competing interests.
