# Supplementary material for: Inadequate Reporting of Cointerventions, Other Methodological Factors, and Treatment Estimates in Cardiovascular Trials: A Meta-Epidemiological Study
Source: Mayo Clin Proc Innov Qual Outcomes. 2023 Jun 2;7(4):231–40. doi: 10.1016/j.mayocpiqo.2023.04.010 (PMC10250579; doi:10.1016/j.mayocpiqo.2023.04.010)
Supplement: Supplemental Data [file mmc1.docx]

**Supplementary**

# Supplementary Figure 1: Prisma flow diagram

Full-text articles assessed for eligibility
(n = 200)

Records screened
(n = 1901)

Records after duplicates removed
(n = 1901)

Additional records identified through hand search
(n = 10)

Records identified through database searching
n = 1606 (Medline) & 812 (Embase)

# Supplementary Figure 2. Forest plot on the association of risk of bias due to deviations of intended interventions and treatment effects (n=162)^a^
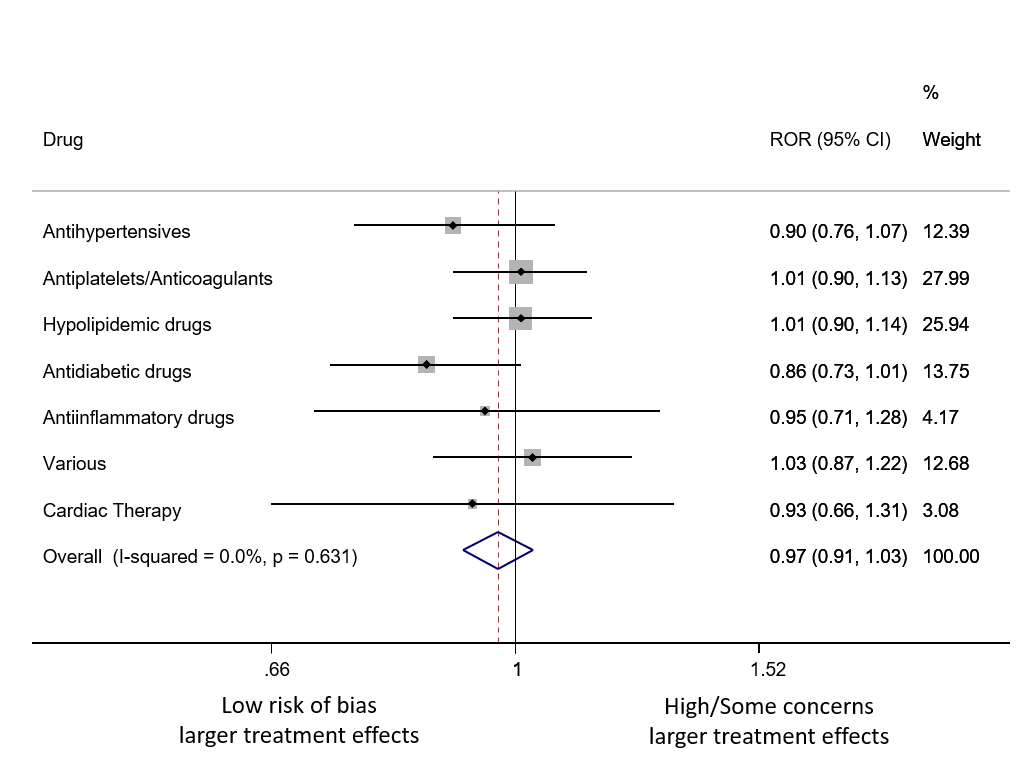


## Included

## Eligibility

## Screening

## Identification

Full-text articles excluded, with reasons:
Not RCT n = 22 Other outcomes n = 13 Other comparison n = 1

Studies included in quantitative synthesis
(n = 164)

Records excluded based on title and abstract
(n = 1701)

Ratio of Odds Ratio (ROR) >1.0 indicating larger treatment effects in studies judged as “at risk of bias” (high/some concerns). **^a^**2 trials did not provide number of events for primary outcome and were therefore excluded.

# Supplementary Figure 3. Forest plot on the association of industry vs. non-industry funding with treatment effects (n=162)^a^


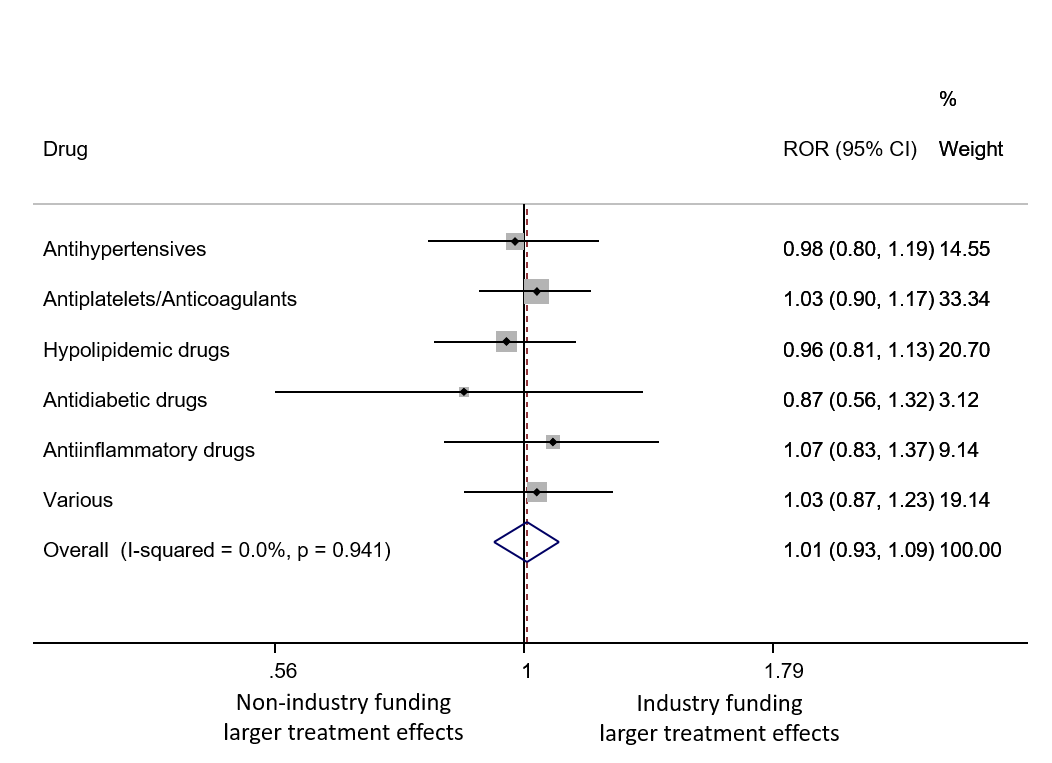


Ratio of Odds Ratio (ROR) >1.0 indicating larger treatment effects in industry-funded trials. **^a^**2 trials did not provide number of events for primary outcome and were therefore excluded.

# Supplementary Table 1. All included RCTs with the corresponding effect sizes as reported in the original publication and Odds Ratio as calculated through random effect meta-analysis, categorized according to adequate reporting of co-intervention vs not

| PMID | Odds Ratio | 95%CI | Reported Effect Size |
| --- | --- | --- | --- |
| **Reporting co-interventions:yes** |  |  |  |
| 23473369 | 0.788 | 0.666 0.933 | adjOR 0.78; 0.66 to 0.93 |
| 25002178 | 1.573 | 1.094 2.260 | Relative Risk 1·52, 1·09-2·13 |
| 24679062 | 0.991 | 0.850 1.155 | HR 0.99, 0.86-1.15 |
| 24679061 | 1.087 | 0.933 1.267 | HR 1.08, 0.93-1.26 |
| 25775052 | 0.640 | 0.458 0.893 | Relative Risk 0.67, 0.50-0.90 |
| 22077909 | 0.987 | 0.729 1.335 | Relative Risk 0.99, 0.74-1.32 |
| 21856483 | 0.765 | 0.577 1.015 | Relative Risk 0.83, 0.68-1.01 |
| 24171490 | 0.574 | 0.407 0.810 | Relative Risk 0.72, 0.43-0.82 |
| 26095867 | 0.478 | 0.261 0.878 | Risk Difference 0.1 %, -0.6 to 0.8 |
| 26933848 | 0.932 | 0.752 1.155 | Relative Risk 0.94, 0.80-1.12 |
| 27774838 | 0.908 | 0.789 1.057 | Relative Risk 0.92, 0.81-1.05 |
| 26460660 | 1.035 | 0.931 1.151 | Relative Risk 1.03, 0.95-1.11 |
| 23803136 | 0.673 | 0.560 0.810 | HR 0.68, 0.57-0.81 |
| 24247616 | 1.244 | 0.961 1.610 | HR 1.25, 0.97-1.61 |
| 21406646 | 0.996 | 0.569 1.745 | HR 1.01, 0.58-1.76 |
| 29900874 | 0.696 | 0.526 0.921 | HR 0.72, 0.55-0.93 |
| 30146935 | 0.939 | 0.827 1.066 | HR 0.94, 0.84-1.05 |
| 28605603 | 0.905 | 0.773 1.060 | HR 0.91, 0.78-1.06 |
| 27633186 | 0.722 | 0.557 0.935 | HR 0.74, 0.58-0.95 |
| 23992601 | 0.998 | 0.888 1.122 | HR 1.00, 0.89-1.12 |
| 30418475 | 1.035 | 0.897 1.194 | HR 1.02, 0.89-1.17 |
| 21616527 | 1.029 | 0.941 1.126 | HR 1.02, 0.94-1.12 |
| 23126252 | 1.039 | 0.927 1.164 | HR 1.04, 0.93-1.16 |
| 29527974 | 1.047 | 0.890 1.231 | HR 1.03, one-sided 1.23 |
| 22085343 | 1.019 | 0.850 1.222 | HR 1.02, 0.87-1.21 |
| 28910237 | 0.923 | 0.835 1.021 | HR 0.91, 0.83-1.00 |
| 26378978 | 0.849 | 0.727 0.992 | HR 0.86, 0.74-0.99 |
| 22931315 | 0.888 | 0.689 1.144 | HR 0.92, 0.72-1.16 |
| 23726159 | 0.817 | 0.637 1.047 | HR 0.81, 0.64-1.03 |
| 27295427 | 0.858 | 0.763 0.965 | HR 0.87, 0.78-0.97 |
| 26886418 | 0.744 | 0.604 0.915 | HR 0.76, 0.62-0.93 |
| 23656645 | 0.987 | 0.885 1.100 | HR 0.97, 0.88-1.08 |
| 23121374 | 0.959 | 0.846 1.088 | HR 0.93, 0.85-1.02 |
| 22686416 | 1.035 | 0.942 1.138 | HR 1.02, 0.94-1.11 |
| 30146932 | 0.965 | 0.864 1.077 | Rate Ratio 0.97, 0.87-1.08 |
| 30146931 | 0.875 | 0.784 0.977 | Rate ratio 0.88, 0.79-0.97 |
| 31738483 | 0.755 | 0.588 0.969 | HR 0.78, 0.61-0.98 |
| 32966714 | 0.998 | 0.866 1.149 | HR 0.97, 0.85-1.11 |
| 32543684 | 0.650 | 0.464 0.911 | HR 0.66, 0.48-0.92 |
| - 31433919 | 0.980 | 0.826 1.163 | HR 0.98, 0.83-1.15 |
| - 31475799 | 1.373 | 1.091 1.729 | HR 1.36, 1.09-1.70 |
| - 33202219 | 0.965 | 0.800 1.165 | OR 0.97, 0.80-1.17 |
| **Not adequately reporting co-interventions** |  |  |  |
| 23995608 | 0.961 | 0.814 1.135 | Relative Risk 0.99, 0.85-1.16 |
| 27590218 | 0.456 | 0.158 1.316 | OR 0.46, 0.12-1.43 |
| 23117776 | 0.813 | 0.653 1.013 | Relative Risk 0.83, 0.67-1.01 |
| 22452807 | 0.854 | 0.654 1.114 | OR 0.88, 0.66-1.13 |
| 26324049 | 0.941 | 0.810 1.093 | Rate Ratio 0.94, 0.81-1.09 |
| 29525821 | 0.872 | 0.683 1.112 | HR 0.88, 0.69-1.11 |
| 23991622 | 1.022 | 0.831 1.257 | HR 1.02, 0.84-1.25 |
| 22782417 | 1.020 | 0.735 1.414 | OR 1.01, 0.73-1.41 |
| 21732835 | 0.920 | 0.784 1.080 | HR 0.93, 0.80-1.08 |
| 29766750 | 0.749 | 0.587 0.955 | HR 0.75, 0.59-0.95 |
| 27160892 | 0.884 | 0.774 1.010 | HR 0.89, 0.78-1.01 |
| 22082198 | 2.295 | 1.331 3.955 | HR 2.29, 1.34-3.94 |
| 27043082 | 1.161 | 0.902 1.494 | HR 1.16, 0.91-1.47 |
| 28844201 | 0.958 | 0.822 1.116 | HR 0.96, 0.83-1.10 |
| 21316752 | 1.086 | 0.826 1.428 | HR 1.09, 0.84-1.41 |
| 21780946 | 0.943 | 0.795 1.119 | HR 0.95, 0.80-1.11 |
| 24206459 | 1.009 | 0.715 1.424 | HR 0.98, 0.70-1.37 |
| 28304242 | 0.884 | 0.764 1.022 | HR 0.88, 0.76-1.02 |
| 29766772 | 1.077 | 0.864 1.343 | HR 1.07, 0.87-1.33 |
| 26321103 | 1.038 | 0.782 1.377 | OR 1.04, 0.78-1.39 |
| 24177257 | 1.031 | 0.764 1.389 | HR 1.03, 0.77-1.38 |
| 23478743 | 0.918 | 0.734 1.147 | HR 0.93, 0.79-1.09 |
| 27959713 | 0.553 | 0.350 0.874 | HR 0.59, 0.47-0.76 |
| 22550196 | 0.580 | 0.329 1.025 | RR 0.78, 0.60-1.03 |
| 21309657 | 0.438 | 0.314 0.613 | HR 0.45, 0.32-0.62 |
| 28402745 | 1.039 | 0.846 1.277 | HR 1.03, 0.85-1,25 |
| 22077816 | 0.923 | 0.841 1.013 | HR 0.92, 0.85-1.01 |
| 22920930 | 0.952 | 0.846 1.072 | HR 0.91, 0.79-1.05 |
| 29544699 | 1.129 | 0.782 1.632 | HR 1.13, 0.79-1.62 |
| 30279197 | 0.717 | 0.402 1.278 | HR 0.73, 0.41-1.27 |
| 23992602 | 0.952 | 0.805 1.125 | HR 0.96, one-sided 1.16 |
| 30291013 | 0.774 | 0.667 0.898 | HR 0.78, 0.68-0.90 |
| 21073363 | 0.638 | 0.531 0.766 | HR 0.63, 0.54-0.74 |
| 26474810 | 0.900 | 0.757 1.070 | HR 0.95, 0.82-1.10 |
| 21870978 | 0.792 | 0.659 0.951 | HR 0.79, 0.66-0.95 |
| 28844192 | 0.752 | 0.655 0.862 | HR 0.76, 0.66-0.86 |
| 21830957 | 0.875 | 0.741 1.035 | HR 0.88, 0.74-1.03 |
| 27367876 | 0.961 | 0.630 1.464 | HR 0.99, 0.76-1.27 |
| 24682069 | 0.949 | 0.812 1.109 | HR 0.96, 0.83-1.11 |
| 30166073 | 0.867 | 0.741 1.014 | Rate Ratio 0.87, 0.75-1.01 |
| 26630143 | 1.020 | 0.880 1.183 | HR 1.02, 0.89-1.17 |
| 28514624 | 1.007 | 0.906 1.121 | HR 1.01, 0.91-1.11 |
| 28304224 | 0.844 | 0.782 0.912 | HR 0.85, 0.79-0.92 |
| 25176015 | 0.774 | 0.700 0.855 | HR 0.80, 0.73-0.87 |
| 30415610 | 0.970 | 0.792 1.188 | HR 0.96, 0.79-1.16 |
| 25176136 | 1.076 | 0.960 1.206 | HR 1.08, 0.96-1.20 |
| 26954408 | 0.957 | 0.742 1.236 | HR 0.88, 0.57-1.34 |
| 23473338 | 1.050 | 0.891 1.238 | HR 1.01, 0.90-1.13 |
| 24251359 | 0.873 | 0.744 1.024 | HR 0.87, 0.73-1.04 |
| 25399658 | 0.717 | 0.597 0.860 | HR 0.71, 0.59-0.85 |
| 22443427 | 0.863 | 0.791 0.942 | HR 0.87, 0.80-0.94 |
| 25173516 | 0.994 | 0.900 1.098 | HR 1.00, 0.91-1.09 |
| 22077192 | 0.821 | 0.719 0.938 | HR 0.84, 0.74-0.96 |
| 25781440 | 0.866 | 0.538 1.395 | HR 0.92, 0.57-1.47 |
| 23121378 | 1.089 | 0.975 1.217 | HR 1.08, 0.98-1.20 |
| 25773268 | 0.844 | 0.745 0.956 | HR 0.85, 0.75-0.96 |
| 30403574 | 0.843 | 0.768 0.926 | HR 0.85, 0.78-0.93 |
| 27959716 | 0.922 | 0.754 1.127 | HR 0.93, 0.76-1.13 |
| 26052984 | 0.985 | 0.890 1.090 | HR 0.98, 0.88-1.09 |
| 27043774 | 0.966 | 0.856 1.090 | HR 0.93, 0.85-1.03 |
| 26551272 | 0.750 | 0.631 0.890 | HR 0.75, 0.64-0.89 |
| 30145941 | 0.986 | 0.849 1.144 | HR 0.99, 0.85-1.14 |
| 24716680 | 0.892 | 0.754 1.056 | HR 0.89, 0.77-1.04 |
| 22551105 | 0.947 | 0.788 1.138 | HR 0.93, 0.79-1.10 |
| 28605608 | 0.850 | 0.673 1.073 | HR 0.86, 0.75-0.97 |
| 28845751 | 0.870 | 0.776 0.976 | HR 0.88, 0.79-0.97 |
| 24678955 | 0.930 | 0.838 1.031 | HR 0.94, 0.85-1.03 |
| 25014686 | 0.959 | 0.893 1.030 | Rate Ratio 0.96, 0.90-1.03 |
| 30535217 | 1.231 | 0.894 1.694 | HR 1.25, 0.94-1.67 |
| 21388310 | 0.994 | 0.899 1.100 | HR 0.99, 0.91-1.08 |
| 28847206 | 0.899 | 0.837 0.965 | Rate Ratio 0.91, 0.85-0.97 |
| 30415602 | 0.832 | 0.728 0.951 | HR 0.83, 0.73-0.95 |
| 25771069 | 0.789 | 0.673 0.925 | HR 0.79, 0.68- 0.93 |
| 24490264 | 0.878 | 0.711 1.085 | HR 0.89, 0.75-1.07 |
| 23532240 | 0.838 | 0.679 1.035 | HR 0.82, 0.69-0.99 |
| 30415628 | 0.737 | 0.661 0.823 | HR 0.75, 0.68-0.83 |
| 21663949 | 0.824 | 0.728 0.933 | Risk Ratio 0.83, 0.74-0.94 |
| 30158069 | 0.956 | 0.806 1.135 | HR 0.96, 0.81-1.13 |
| 25401325 | 0.934 | 0.765 1.139 | HR 0.94, 0.77-1.15 |
| 26323937 | 0.926 | 0.787 1.090 | HR 0.93, 0.81-1.07 |
| 27041480 | 0.928 | 0.781 1.103 | HR 0.93, 0.79-1.10 |
| 27039945 | 0.707 | 0.552 0.905 | HR 0.71, 0.56-0.90 |
| 27040132 | 0.762 | 0.641 0.907 | HR 0.76, 0.64-0.91 |
| 26039521 | 0.915 | 0.858 0.975 | HR 0.94, 0.89-0.99 |
| 22686415 | 0.982 | 0.870 1.109 | HR 0.98, 0.87-1.10 |
| 30043065 | 0.599 | 0.379 0.946 | HR 0.69, 0.49- 0.96 |
| 23117775 | 1.028 | 0.930 1.136 | HR 1.01, 0.91-1.10 |
| 32222134 | 0.879 | 0.784 0.986 | HR 0.90, 0.82-0.98 |
| 33200892 | 0.492 | 0.392 0.618 | HR 0.67, 0.52-0.85 |
| 33200891 | 0.735 | 0.641 0.842 | HR 0.74, 0.63-0.88 |
| 32222135 | 0.844 | 0.741 0.961 | HR 0.85, 0.76-0.96 |
| 31479209 | 0.859 | 0.607 1.214 | HR 0.87, 0.62-1.21 |
| 31733180 | 1.382 | 1.013 1.885 | HR 1.35, 1.01-1.81 |
| 31091372 | 0.845 | 0.686 1.040 | HR 0.85, 0.69-1.03 |
| 31189511 | 0.882 | 0.784 0.993 | HR 0.88, 0.79-0.99 |
| 32334703 | 0.806 | 0.615 1.057 | HR 0.71, 0.54-0.94 |
| 32970396 | 0.594 | 0.492 0.718 | HR 0.61, 0.51-0.72 |
| 32668111 | 0.823 | 0.703 0.963 | HR 0.83, 0.71-0.96 |
| 32882163 | 0.684 | 0.509 0.918 | HR 0.70, 0.52-0.92 |
| 31535829 | 0.723 | 0.624 0.838 | HR 0.74, 0.65-0.85 |
| - 33190147 | 0.986 | 0.887 1.095 | HR 0.99, 0.90-1.09 |
| 32865380 | 0.687 | 0.565 0.835 | HR 0.69, 0.57-0.83 |
| 32865374 | 0.342 | 0.190 0.615 | HR 0.34, 0.19-0-61 |
| 31610549 | 2.145 | 0.770 5.975 | Risk Ratio 1.83 [95% CI, 0.71 to 4.76 |
| 32865377 | 0.731 | 0.626 0.854 | HR 0.75, 0.65-0.86 |
| 30990260 | 0.685 | 0.574 0.816 | HR 0.70, 0.59-0.82 |
| 33197395 | 0.529 | 0.415 0.675 | Rate Ratio 0.79, 0.62-1.01 |
| 32219359 | 0.817 | 0.634 1.051 | HR 0.82, 0.65-1.04 |
| 31475794 | 0.808 | 0.720 0.907 | Rate ratio, 0.87, 0.75-1.01 |
| 31475798 | 0.890 | 0.802 0.987 | HR 0.90, 0.81-0.99 |
| 31733140 | 0.762 | 0.602 0.964 | HR 0.77, 0.61-0.96 |
| 33185990 | 0.914 | 0.836 0.999 | HR 0.92, 0.86-0.99 |
| 31475793 | 1.402* | 1.052 1.869 | HR 0.72, 0.55-0.95 |
| 32877651 | 0.979 | 0.869 1.103 | HR 0∙98, 0∙88-1∙09 |
| 33181081 | 0.697 | 0.569 0.854 | HR 0·85, 0·70-1·03 |
| 31448738 | 0.650 | 0.540 0.782 | HR 0·66, 0·55-0·80 |
| 31461120 | 1.174* | 0.748 1.843 | Estimate of difference 0.4 (− to 1.3) |
| 31536101 | 0.976 | 0.835 1.141 | HR, 0.98, 0.84-1.14 |
| 31237644 | 0.627 | 0.408 0.964 | HR 0.64, 0.42-0.98 |
| 31063575 | 0.907 | 0.651 1.265 | HR 0.89, 1-sided 97.5%CI, 0-1.23 |

*outcomes were coded so that an OR less than 1 indicates a beneficial effect of the experimental intervention; 3-arm trials (or other multi-arm trials with more than 2 arms) were included in their literature search but only the results of one experimental intervention vs placebo included in the analysis

# Supplementary Table 2. Literature search.

| (((("Annals of internal medicine"[Journal]) OR ("BMJ (Clinical research ed.)"[Journal]) OR  ("JAMA"[Journal]) OR ("Lancet (London, England)"[Journal]) OR ("The New England journal of  medicine"[Journal])) AND (randomized controlled trial[pt] OR controlled clinical trial[pt] OR  randomized[tiab] OR placebo[tiab] OR drug therapy[sh] OR randomly[tiab] OR trial[tiab] OR groups[tiab] NOT (animals[mh] NOT humans[mh]))) AND (("Cardiovascular Diseases/drug therapy"[Mesh] OR "Cardiovascular Diseases/mortality"[Mesh] OR "Cardiovascular Diseases/prevention and control"[Mesh]) OR ("Myocardial Ischemia/drug therapy"[Mesh] OR "Myocardial Ischemia/mortality"[Mesh] OR "Myocardial Ischemia/prevention and control"[Mesh]) OR ("Myocardial Infarction/drug therapy"[Mesh] OR "Myocardial Infarction/mortality"[Mesh] OR "Myocardial Infarction/prevention and control"[Mesh]) OR ("Stroke/drug therapy"[Mesh] OR "Stroke/mortality"[Mesh] OR "Stroke/prevention and control"[Mesh]) OR ("Cerebrovascular Disorders"[Mesh:noexp]) OR ("Ischemic Attack, Transient"[Mesh]) OR ("Intracranial Embolism and Thrombosis"[Mesh]) OR ("Intracranial Arteriosclerosis"[Mesh:noexp]))) NOT ((comment[Publication Type]) OR (letter[Publication Type])) Filters: Publication date |
| --- |
